# The effect of neuraminidase inhibitors on household transmission in Japanese patients with influenza A and B infection: A prospective, observational study

**DOI:** 10.1111/irv.12590

**Published:** 2018-12-28

**Authors:** Nobuo Hirotsu, Yutaka Saisho, Takahiro Hasegawa

**Affiliations:** ^1^ Hirotsu Clinic Kawasaki Japan; ^2^ Shionogi & Co., Ltd Osaka Japan

**Keywords:** household secondary infection, influenza transmission, laninamivir, oseltamivir, peramivir, zanamivir

## Abstract

**Background:**

The relative ability of neuraminidase inhibitors (NAIs) to reduce household influenza transmission when given to index patients is not established.

**Objectives:**

To compare daily secondary infection rates (SIR) of influenza A (A/H1pdm and A/H3) and B in households of index patients treated with oseltamivir, zanamivir, laninamivir, or peramivir.

**Patients/Methods:**

This Japanese, single‐center, prospective, observational study (UMIN‐CTR: UMIN000024650) enrolled index patients with confirmed influenza who were treated with an NAI during 6 influenza seasons (2010‐2016). Secondary infection patients were household members diagnosed with the same influenza subtype 1‐7 days after onset in the index patient. Daily SIR was calculated using a modified Reed‐Frost model. The rate of household members with secondary infection and proportion of households with any secondary infection were also calculated.

**Results:**

Index patients with influenza A (n = 1146) or B (n = 661) were enrolled (~3400 total index and secondary patients). Daily SIR for all virus subtypes was highest when oseltamivir was used (eg, unadjusted estimate: type A, 1.47% vs 0.71%‐1.13%; type B, 1.30% vs 0.59%‐0.88%). Pairwise comparisons revealed significant differences in daily SIR between NAIs for influenza type A, type B, and subtype A/H3; for example, for type A, SIR was significantly higher with oseltamivir than with peramivir or zanamivir. The rate of household members with secondary infection and proportion of households with any secondary infection also varied between NAIs.

**Conclusions:**

Neuraminidase inhibitors differed in their ability to reduce household influenza transmission; transmission was highest with oseltamivir. Physicians may consider effects on household transmission when deciding which NAI to prescribe.

## 1 INTRODUCTION

Neuraminidase inhibitors (NAIs) reduce the duration and severity of illness caused by influenza in both adults and children, although the effects may be modest because NAIs inhibit viral replication by interfering with the release of virus from infected cells, but are not virucidal.^1^, ^2^, ^3^ NAIs are effective when administered within 48 hours of the onset of influenza symptoms^2^ and can reduce the rate of secondary infection when used prophylactically by asymptomatic people who are in close contact with an infected person (eg, household members).^1^, ^3^, ^4^ In Japan, unlike in most other countries, NAIs are mainly prescribed for outpatients.^5^ The Japanese health insurance system covers the cost of rapid influenza diagnostic tests (RIDTs), and more than 80% of Japanese patients with influenza visit a medical clinic within 48 hours of onset, greatly facilitating the early diagnosis and treatment of influenza.^5^ NAIs currently approved in Japan for the treatment of influenza include oral oseltamivir, inhaled zanamivir, inhaled laninamivir, and intravenous peramivir.

Reduction in the household transmission of influenza is critical in reducing the overall public health effects of this infectious disease. Several studies suggest that NAI treatment of the primary (index) infected patient may reduce household transmission of influenza without the need for prophylaxis of uninfected individuals.^6^, ^7^, ^8^, ^9^, ^10^, ^11^, ^12^, ^13^ Effective reduction in transmission by treatment of the index patient would avoid the logistic and cost implications of widespread prophylactic use of NAIs. Influenza transmission is related to the extent and duration of virus shedding, which varies between influenza subtypes^14^ and may be reduced by NAI treatment.^7^, ^11^, ^15^ We recently conducted a randomized controlled trial examining the effect of NAIs on virus clearance in children aged 4‐12 years with influenza A infection.^16^ The time required for clearance of influenza virus was significantly shorter in children treated with peramivir than in those treated with oseltamivir (median time to clearance 2.05 vs 3.08 days, adjusted *P *=* *0.0348). Differences in the ability of NAIs to reduce viral shedding or accelerate viral clearance may translate into different effectiveness in reducing transmission. Previous studies comparing the ability of different NAIs to reduce household transmission have led to varying results.^6^, ^8^, ^9^, ^10^ However, these studies have been limited by small sample size, number of NAIs used, and/or retrospective design (eg, claims database). Further, none of the studies separately analyzed transmission of both influenza A (including subtypes) and influenza B, nor did any include the more recently available NAI, peramivir.

The primary objective of this prospective, observational, household transmission study was to compare the daily secondary infection rate (SIR) in households of index patients treated with one of four NAIs available in Japan (oseltamivir, zanamivir, laninamivir, and peramivir). Secondary objectives included the effect of different NAIs on the rate of household members with secondary infection and the proportion of households with at least one secondary infection.

## 2 MATERIALS AND METHODS

### 2.1 Study design

This was a prospective household transmission study that enrolled patients with confirmed influenza who attended the Hirotsu Clinic (Kawasaki, Japan) for treatment with an NAI during 6 Northern Hemisphere influenza seasons between 2010 and 2016. The trial is registered at UMIN‐CTR (number UMIN000024650), and the protocol was approved by the ethics committee of Shionogi & Co., Ltd. Informed consent was obtained using an opt‐out procedure, in which study information was posted within the clinic, and patients were provided with opportunities to decline participation. To minimize potential selection bias and other biases, a third‐party vendor (Medical TOUKEI Corporation, Tokyo, Japan), selected and funded by Shionogi & Co., Ltd, derived the analysis dataset from the study database, obtained from medical records of the Hirotsu Clinic. The vendor was responsible for data anonymization and dataset optimization for analysis, and also verified all analyses conducted by Shionogi & Co., Ltd. The study database was audited by IBEC Co., Ltd. (Osaka, Japan).

### 2.2 Study population

**Figure 1:**
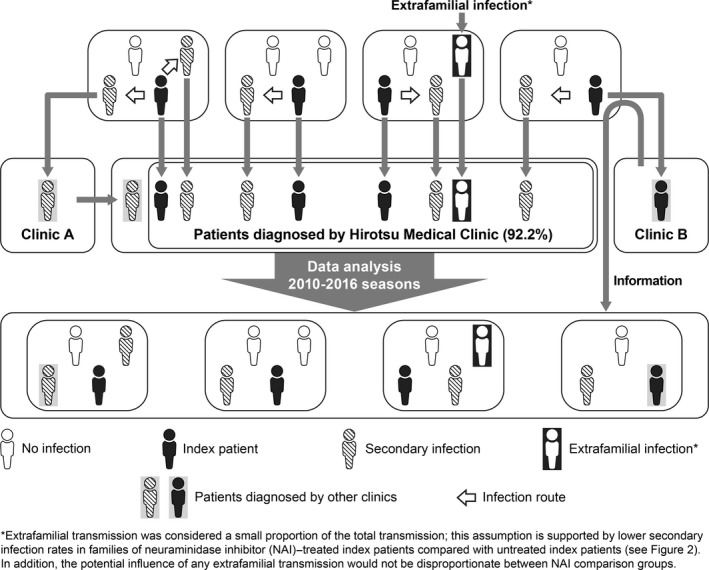
Schematic depicting the source of index and secondary infection patients included in this analysis

Patients of any age who attended the Hirotsu Clinic and were diagnosed with influenza A (subtypes A/H1pdm or A/H3) or B using RIDTs were eligible for inclusion. ImmunoAce^®^ Flu (Tauns Laboratories, Inc., Shizuoka, Japan) was used for differential diagnosis of influenza A and B, and LineJudge^®^ (Tauns Laboratories, Inc.) was used for differentiation of influenza A H1pdm and H3 subtypes. As this was an observational study, patients were treated with an NAI (oseltamivir, zanamivir, laninamivir, or peramivir), or remained untreated (eg, if patient visited clinic >48 hours after onset of illness), at the discretion of the physician and in consideration of factors such as age, influenza type and severity, and expected level of adherence. The dosage and administration of NAIs were as per the package insert for each product. Secondary infection patients were household members who were diagnosed with the same influenza type/subtype as the index patient between 24 hours and 7 days after the onset of symptoms in the index patient (Figure 1), similar to the time frame used in previous studies of influenza household transmission in Japan.^9^, ^12^, ^17^ Extrafamilial transmission was considered to be a small proportion of the total and randomly distributed among the families regardless of NAI used to treat the index patient. There was no prophylactic NAI treatment of family members. Almost all (92.2%) patients (index and secondary infection) attended the Hirotsu Clinic on their first visit (Figure 1). For the other 7.8% of patients, confirmation of influenza diagnosis and other information was obtained when the patient attended the Hirotsu Clinic on a subsequent visit, from other patients in that household who attended the Hirotsu Clinic, or from the other clinic.

### 2.3 Data collection

The following information was collected, from medical records held at Hirotsu Clinic (or, in a few cases, from other clinics), patient diaries, or direct questioning, for index patients who agreed to participate in the study: age; gender; history of influenza and influenza vaccination (previous and current season); current (and previous, if applicable) virus type/subtype; dates of symptom and fever onset and disappearance; type and date(s) of NAI medication; body temperature, measured at first visit and 4 times daily until alleviation of fever; family composition (at any clinic visit during the survey year; confirmed by index patients with permission from family members); and the clinical course of influenza. Patients were instructed on how to record body temperature, signs and symptoms, medication, etc., in a diary provided at the time of first administration of an NAI. Additional information was obtained by a follow‐up phone call or recorded in the diary by patients.

### 2.4 Outcome measures

The primary outcome was the daily SIR, defined as the probability of passing influenza virus to an uninfected household member per day. Secondary outcomes included the rate of household members with secondary infection, defined as the number of infected household members divided by the total number of uninfected household members at baseline (ie, onset time of influenza symptoms in the index patient), and the proportion of households with secondary infection patients, defined as the number of households with at least one secondary infection divided by the total number of households with any uninfected household members at baseline.

### 2.5 Statistical analysis

The analysis population included index patients and secondary infection patients within households. Patients who had infection in multiple seasons were considered as different patients. Summary statistics of the demographic characteristics of index patients were calculated by the influenza subtype (A, A/H1pdm, A/H3, and B) and by the NAI used.

For the primary analysis, the daily SIR was estimated by the influenza subtype and by NAI, as well as for the untreated period (*p*_*0*_) before the start of NAI treatment. A Reed‐Frost model modified to consider the infectious period and the time‐dependent treatment was applied to each family (Figure S1, Table S1). The model also accounted for the possibility that any third or later infection within a family could have been acquired from any of the previous infections (ie, not only from the primary infection). All pairwise comparisons of the daily SIRs among the four NAIs were performed using the modified Reed‐Frost model, after adjusting for the following covariates of patients with infectious ability in a household using the logit‐link function: age, time from onset to start of treatment, and influenza vaccine in the same season. This covariate adjustment was used to minimize any effect of selection bias related to the non‐randomized prescription of NAIs. Sensitivity analyses were performed based on the period with infectious ability defined by the combination of time from onset (within 4, 5, 6, 7, or 8 days after confirming a primary infection) and time from disappearance of fever (until 2, 3, or 4 days after disappearance of fever in a primary infection).

Secondary analyses were conducted by the influenza subtype and by the NAI used to treat the index patient. The rate of household members with secondary infections was compared among the four NAIs using a Poisson regression model with an offset parameter of the number of uninfected household members at baseline (Table S1). The proportion of households with secondary infection patients was compared among the four NAIs using a logistic regression model. Sensitivity analyses were performed using the same methods as for the primary analysis.

For all analyses, a two‐sided test with statistical significance level of 0.05 was used. There was no imputation for missing data, no adjustment for multiplicity due to the exploratory nature of the analysis, and no outliers were excluded. All statistical analyses were performed using SAS software, version 9.4 (SAS Institute, Cary, NC, USA).

## 3 RESULTS

### 3.1 Demographic characteristics of index patients

A total of 1807 index patients were identified, most of whom had influenza A infections (Table 1; Table S2). Although most index patients were relatively young, the range of ages was broad in all study groups. Approximately 16% of index patients reported having had influenza during the previous season. Approximately 50% of index patients reported having influenza vaccination in the previous season, and a similar percentage reported vaccination in the current season. Most index patients started NAI treatment within 2 days of disease onset, with the mean and median <1 day for most study groups. Peramivir and oseltamivir were the most commonly prescribed NAIs for influenza A, and zanamivir was the most commonly prescribed NAI for influenza B. Laninamivir was the least commonly used NAI, and a small number of patients were untreated. There were approximately 3 (mean) uninfected people per household at the onset of the primary infection. Approximately 3400 patients (both index and secondary patients) from 1200 families were included in the analysis.

**Table 1:** Demographic characteristics of index patients infected with influenza A or B

Influenza type	Variable	Neuraminidase treatment				
		Peramivir	Oseltamivir	Zanamivir	Laninamivir	Untreated
A	Number of index patients	378	323	248	170	27
	Season, n (%)					
	2010‐2011	37 (9.8)	62 (19.2)	38 (15.3)	46 (27.1)	6 (22.2)
	2011‐2012	55 (14.6)	52 (16.1)	73 (29.4)	20 (11.8)	4 (14.8)
	2012‐2013	77 (20.4)	64 (19.8)	28 (11.3)	17 (10.0)	3 (11.1)
	2013‐2014	59 (15.6)	39 (12.1)	22 (8.9)	23 (13.5)	4 (14.8)
	2014‐2015	86 (22.8)	51 (15.8)	55 (22.2)	50 (29.4)	7 (25.9)
	2015‐2016	64 (16.9)	55 (17.0)	32 (12.9)	14 (8.2)	3 (11.1)
	Male, n (%)	180 (47.6)	179 (55.4)	110 (44.4)	86 (50.6)	18 (66.7)
	Age, years					
	n	378	323	248	169	26
	Mean (SD)	29.8 (19.0)	12.8 (16.7)	14.0 (11.6)	16.3 (12.8)	19.8 (16.5)
	Median (min., max.)	30.0 (2, 88)	5.0 (0, 69)	10.0 (3, 65)	11.0 (3, 57)	12.0 (0, 47)
	Number of uninfected people per family at onset^a^					
	n	378	323	248	170	27
	Mean (SD)	2.5 (1.1)	2.7 (0.9)	2.8 (1.0)	2.9 (0.9)	3.0 (1.1)
	Median (min., max.)	3.0 (0, 6)	3.0 (0, 6)	3.0 (0, 6)	3.0 (0, 6)	3.0 (1, 5)
	Presence of influenza during previous season, n (%)	36 (9.5)	46 (14.2)	47 (19.0)	42 (24.7)	2 (7.4)
	Influenza vaccination in the previous season, n (%)	205 (54.2)	169 (52.3)	137 (55.2)	94 (55.3)	9 (33.3)
	Influenza vaccination in the same season, n (%)	185 (48.9)	172 (53.3)	115 (46.4)	88 (51.8)	7 (25.9)
	Body temperature at first visit, °C					
	n	378	314	247	167	26
	Mean (SD)	38.11 (0.85)	38.28 (0.88)	38.14 (0.84)	38.09 (0.85)	37.40 (0.88)
	Time from onset to start of treatment, days					
	n	378	322	248	170	NA
	Mean (SD)	0.7 (0.6)	0.8 (0.7)	0.8 (0.7)	0.7 (0.6)	NA
	Median (min., max.)	0.6 (0, 4)	0.7 (0, 4)	0.7 (0, 3)	0.7 (0, 3)	NA
	Time from start of treatment to disappearance of fever, days					
	n	335	298	241	159	NA
	Mean (SD)	0.8 (0.7)	1.1 (1.0)	1.1 (1.1)	1.1 (1.0)	NA
	Median (min., max.)	0.8 (−2, 5)	0.8 (−2, 6)	0.9 (−1, 6)	0.8 (−1, 6)	NA
B	Number of index patients	100	95	360	85	21
	Season, n (%)					
	2010‐2011	5 (5.0)	15 (15.8)	39 (10.8)	21 (24.7)	6 (28.6)
	2011‐2012	16 (16.0)	17 (17.9)	119 (33.1)	21 (24.7)	4 (19.0)
	2012‐2013	2 (2.0)	0	5 (1.4)	1 (1.2)	0
	2013‐2014	33 (33.0)	31 (32.6)	131 (36.4)	28 (32.9)	4 (19.0)
	2014‐2015	5 (5.0)	1 (1.1)	4 (1.1)	1 (1.2)	2 (9.5)
	2015‐2016	39 (39.0)	31 (32.6)	62 (17.2)	13 (15.3)	5 (23.8)
	Male, n (%)	42 (42.0)	46 (48.4)	174 (48.3)	43 (50.6)	6 (28.6)
	Age, years					
	n	100	95	360	85	21
	Mean (SD)	20.4 (17.9)	6.7 (7.5)	12.2 (12.3)	16.5 (13.2)	14.3 (11.5)
	Median (min., max.)	12.5 (2, 82)	5.0 (0, 58)	8.0 (3, 84)	11.0 (6, 74)	9.0 (3, 41)
	Number of uninfected people per family at onset^a^					
	n	100	95	360	85	21
	Mean (SD)	2.7 (1.2)	2.8 (0.7)	2.8 (0.9)	2.9 (0.9)	2.6 (1.3)
	Median (min., max.)	3.0 (0, 6)	3.0 (1, 5)	3.0 (0, 6)	3.0 (0, 5)	3.0 (0, 5)
	Presence of influenza during previous season, n (%)	11 (11.0)	20 (21.1)	60 (16.7)	20 (23.5)	3 (14.3)
	Influenza vaccination in the previous season, n (%)	67 (67.0)	55 (57.9)	196 (54.4)	42 (49.4)	8 (38.1)
	Influenza vaccination in the same season, n (%)	49 (49.0)	56 (58.9)	188 (52.2)	34 (40.0)	7 (33.3)
	Body temperature at first visit, °C					
	n	100	93	359	83	20
	Mean (SD)	38.23 (0.85)	38.47 (0.87)	38.12 (0.75)	37.97 (0.80)	37.74 (0.99)
	Time from onset to start of treatment, days					
	n	100	95	360	85	NA
	Mean (SD)	0.8 (0.7)	0.8 (0.7)	1.0 (0.8)	1.1 (0.9)	NA
	Median (min., max.)	0.6 (0, 3)	0.6 (0, 4)	0.8 (0, 4)	0.9 (0, 3)	NA
	Time from start of treatment to disappearance of fever, days					
	n	88	88	340	77	NA
	Mean (SD)	1.5 (1.1)	2.0 (1.4)	1.7 (1.3)	1.3 (1.1)	NA
	Median (min., max.)	1.5 (0, 5)	1.8 (−2, 6)	1.5 (−1, 7)	1.0 (−1, 4)	NA

max., maximum; min., minimum; NA, not applicable; SD, standard deviation.

a Not including index patient.

### 3.2 Daily secondary infection rate

**Figure 2:**
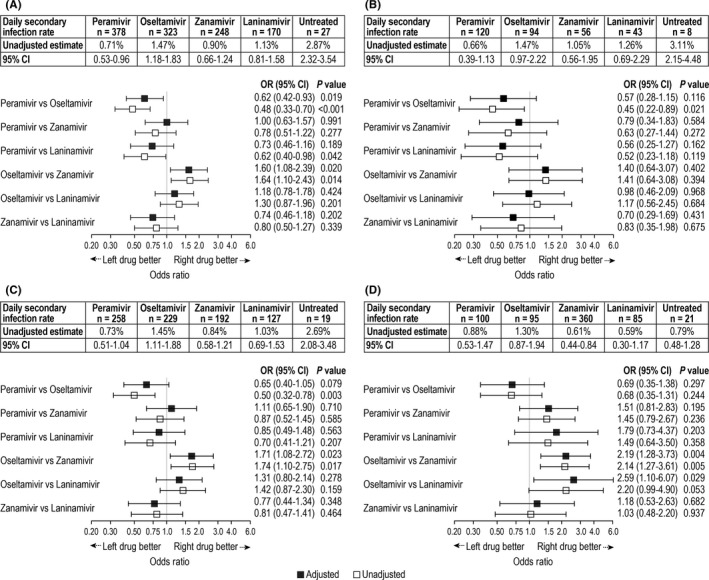
Daily household secondary infection rates for influenza A (panel A), A/H1pdm (panel B), A/H3 (panel C), and B (panel D). Shown are unadjusted estimates and 95% confidence intervals (CI) when patients (index and secondary infection) were treated with peramivir, oseltamivir, zanamivir, or laninamivir, or when patients were untreated, and unadjusted and adjusted odds ratios (OR) of pairwise comparisons between neuraminidase inhibitors

The daily SIR differed according to the NAI used by index and secondary infection patients (Figure 2). Daily SIRs for all influenza subtypes were highest for patients treated with oseltamivir compared with other NAIs. Pairwise comparisons of the daily SIR indicated that household transmission of influenza A was lower with peramivir or zanamivir than with oseltamivir (Figure 2A). This difference was primarily due to differences in reducing transmission of subtype A/H3; although after adjusting for covariates, statistical significance was reached only for the zanamivir vs oseltamivir comparison (Figure 2C). Transmission of influenza B was also lower with zanamivir or laninamivir than with oseltamivir (Figure 2D). Compared with no treatment, all NAIs reduced the daily SIR of influenza A, with the extent of daily SIR reduction ranging from 49% (reduced from 2.87% to 1.47%) with oseltamivir to 75% (reduced from 2.87% to 0.71%) with peramivir. The results of sensitivity analyses were similar (data not shown).

### 3.3 Rate of household members with secondary infections

**Figure 3:**
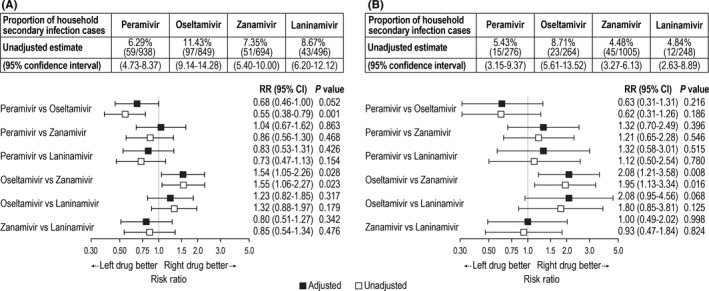
Rate of household secondary infections for influenza A (panel A) and B (panel B). Shown are unadjusted estimates and 95% confidence intervals (CI) when index patients were treated with peramivir, oseltamivir, zanamivir, or laninamivir, and unadjusted and adjusted risk ratios (RR) of pairwise comparisons between neuraminidase inhibitors

As with the daily SIR, the overall rate of household secondary infection varied depending on the NAI used by the index patient (Figure 3). Regardless of the NAI used, the rate of household secondary infection was higher for influenza A than for influenza B. The highest unadjusted rate of household secondary infection with either influenza A or B occurred when the index patient was treated with oseltamivir. After adjusting for covariates, household transmission of both influenza A and B was significantly lower with zanamivir than with oseltamivir. Influenza A transmission also appeared to be lower with peramivir than with oseltamivir, but the difference did not reach statistical significance. Sensitivity analyses yielded similar results (data not shown).

### 3.4 Proportion of households with secondary infections

**Figure 4:**
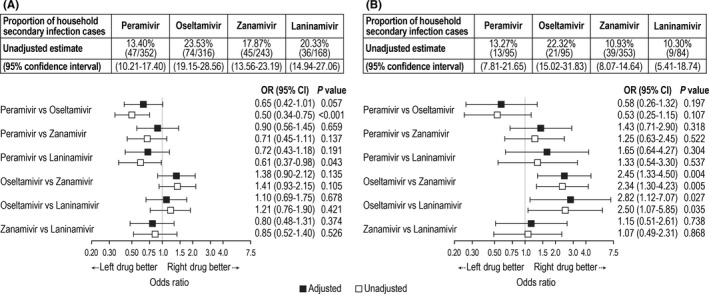
Proportion of households with any secondary infection for influenza A (panel A) and B (panel B). Shown are unadjusted estimates and 95% confidence intervals (CI) when index patients were treated with peramivir, oseltamivir, zanamivir, or laninamivir, and unadjusted and adjusted odds ratios (OR) of pairwise comparisons between neuraminidase inhibitors

The proportion of households with at least one secondary infection also varied depending on the NAI used by the index patient (Figure 4). The highest unadjusted proportion of households with secondary infection occurred when the index patient was treated with oseltamivir, particularly for influenza B. After adjusting for covariates, household transmission of influenza B, but not influenza A, was significantly lower with zanamivir or laninamivir than with oseltamivir. Influenza A transmission also appeared to be lower with peramivir than with oseltamivir, but the difference did not reach statistical significance. The results of sensitivity analyses were similar (data not shown).

## 4 DISCUSSION

In this large, prospective, observational study of data collected across six influenza seasons, the rate of influenza transmission within households varied depending on the NAI used to treat the index and secondary infection patients. Although all NAIs reduced the daily SIR of influenza A compared with no treatment, pairwise comparisons revealed significant differences between NAIs in the extent of reduction in household transmission. Differences between NAIs were also observed for the daily SIR of influenza type B, as well as for the rate of household members who became infected and the proportion of households with any secondary infection. This is the first study to compare the effect of four currently approved NAIs on household transmission of influenza, and the first to analyze transmission according to viral type and subtype. Importantly, adjusting for covariates (eg, patient age) minimized biases related to the choice of NAI, allowing us to compare the ability of NAIs to reduce household transmission in this non‐randomized study. Our results have important implications for which NAI doctors choose when treating patients, as some NAIs may minimize secondary infections within households more than other NAIs. Reduction in household transmission will greatly improve efforts to limit the spread of influenza, especially during epidemic or pandemic conditions.

Although several previous studies have compared the ability of NAIs to reduce household transmission, this is the first to compare all four NAIs available in Japan, the first to examine influenza types and subtypes, and the first to include data from more than one season. Consistent with our study, two previous studies reported that influenza transmission was higher with oseltamivir than with zanamivir^9^, ^10^ or laninamivir,^10^ although a third study found no difference between oseltamivir and zanamivir.^6^ All these studies focused on a single influenza season, and none characterized transmission by influenza type or subtype. Further, no previous study has included intravenous peramivir in the comparison, probably because it is prescribed to outpatients less often than other NAIs due to its route of administration.^5^ The current study also indicates that peramivir generally shows a lower daily SIR and is similar to zanamivir in reducing household transmission of influenza A, the dominant influenza type in almost all seasons. In the other type and subtype analyses, the differences in daily SIR between NAIs were most apparent for influenza subtype A/H3 and influenza B, whereas the ability to reduce household transmission of influenza A/H1pdm did not differ significantly. Apart from oseltamivir, we found no significant differences between the other NAIs in their ability to limit household transmission. Although oseltamivir‐resistant strains of influenza A were prevalent before the appearance of the 2009 pandemic A/H1N1 subtype,^18^ these strains now account for a small percentage of circulating virus in Japan^19^ and are unlikely to explain the lower efficacy of oseltamivir we observed in the 2010‐2016 seasons.^18^

The differences between NAIs in the ability to reduce household influenza transmission may relate to differential effects on viral dynamics. NAI treatment reduces the duration of viral shedding, especially when administered within 48 hours after the onset of illness.^11^, ^20^, ^21^ This reduction in viral shedding may lessen household transmission, particularly if given early.^9^ Several studies have compared the ability of oseltamivir to reduce viral shedding with that of other NAIs.^14^, ^22^, ^23^, ^24^ Two of these studies included patients infected with oseltamivir‐resistant strains of A/H1 that were prevalent before the emergence of the oseltamivir‐susceptible 2009 pandemic strain,^18^ making it difficult to compare with our A/H1pdm results.^22^, ^24^ However, these studies reported no difference between oseltamivir and laninamivir on shedding of influenza A/H3^22^, ^24^ or B.^22^ A more recent study found no difference between oseltamivir, laninamivir, or peramivir in the proportion of patients with viral shedding of both A/H1pdm and A/H3 subtypes.^14^ In contrast, shedding at Day 7 was significantly lower in patients with A/H1, A/H3, or B infections treated with zanamivir than in those treated with oseltamivir, although there were no differences at Days 3‐4.^23^ NAIs may also differ in their ability to reduce viral load, which may, in turn, reduce household transmission. We recently conducted a randomized trial in which peramivir led to significantly more rapid viral clearance (ie, time to first undetectable virus titer) than oseltamivir in children aged 4‐12 years who were mostly infected with A/H3.^16^ In contrast, the time to resolution of fever or symptoms did not vary with NAI treatment.^16^ Other randomized trials have also reported that viral load is reduced more rapidly with peramivir than with oseltamivir, although the reported differences varied and were not always statistically significant.^25^, ^26^, ^27^ Although our data do not allow us to directly link the efficacy of different NAIs on viral shedding or clearance with their effects on household transmission, we hypothesize that NAIs that decrease viral load more rapidly may be more effective at reducing household transmission than slower‐acting NAIs, particularly if initiated within 24 hours after illness onset.

This study is strengthened by the large sample size of index and secondary infection patients of any age, inclusion of data from six influenza seasons, and the analysis of four NAIs and several influenza types and subtypes. In addition, we used a novel modification of the Reed‐Frost model that accounts for the possibility that any third or later infection within a family could have been transmitted from any of the previously infected family members. Statistical analysis models in previous studies were limited by the assumption that subsequent infections within a family were always transmitted from the primary infection or the models did not consider this aspect at all.^6^, ^7^, ^9^, ^10^, ^11^, ^12^, ^13^

Although this study is limited by its observational, non‐randomized design, the results do reflect real‐world clinical practice, albeit in a single clinic in a major Japanese city. There is potential for bias in the choice of NAI for specific patients, for example, for children vs adults or for patients who cannot use inhaled NAIs. However, such potential bias would have been minimized by adjusting for age in the analysis. There is also potential bias if patients with more severe symptoms, which may reflect a more transmissible infection, were more likely to visit the clinic, and to visit soon after the onset of symptoms, than patients with milder symptoms. However, body temperatures at first visit were only moderately elevated (approximately 38°C), suggesting that symptoms were generally not severe. As with all household transmission studies, asymptomatic household members (both primary and secondary infections) could not be identified for inclusion. In addition, the relatively small number of patients with influenza A/H1pdm and influenza B may have limited our ability to detect a significant difference between NAIs for these strains of virus. Further, we did not analyze serial intervals or adherence rates for NAIs requiring multiple doses, although patients were followed closely and were assumed to have completed the prescribed course. In practice, better adherence would be expected with single‐dose NAIs such as peramivir than with multidose NAIs. Finally, although our analysis of daily SIR adjusted for age, time from onset to start of treatment, and vaccine in the same season, other factors may have affected the results.

In conclusion, NAIs differ in their ability to reduce household transmission of influenza, with oseltamivir being generally less effective than other NAIs, particularly peramivir and zanamivir. Given the public health implications of limiting the spread of influenza infection, physicians should consider prescribing NAIs that are most effective at reducing household transmission and ensure that treatment is initiated early.

## CONFLICTS OF INTEREST

YS and TH are employees of Shionogi & Co., Ltd. NH has received research funding and has served as a consultant, advisory board member, and/or speaker for Shionogi & Co., Ltd.

## AUTHOR CONTRIBUTIONS

All authors participated in the study design and interpretation of study results, and in the drafting, critical revision, and approval of the final version of the manuscript. NH was an investigator in the study and was responsible for data collection. TH conducted the statistical analysis.
