# Supplementary material for: The effect of neuraminidase inhibitors on household transmission in Japanese patients with influenza A and B infection: A prospective, observational study
Source: Influenza Other Respir Viruses. 2018 Dec 28;13(2):123–32. doi: 10.1111/irv.12590 (PMC6379638; doi:10.1111/irv.12590)
Supplement: Supplementary file 1 [file IRV-13-123-s001.docx]

supporting information

**Figure S1.** Modified Reed-Frost model.


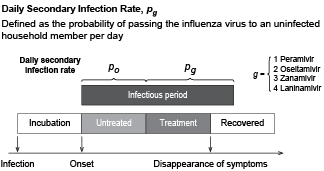


| - *p_0_* = probability of passing influenza virus during untreated period - *p_g_* = probability of passing influenza virus after start of neuraminidase inhibitor treatment - Time point *t* = 0 (day of onset in index patient), 1, …, *T* - Infectious period: *H* - In household *k* (*k* = 1, …, *K*)   - Number of uninfected members at time 0: *n_k_*   - Number of uninfected members at time *t*: *S_kt_*   - Number of onset patients at time *t*: *E_kt_*   *S_kt_* = *S_k_*_,_*_t_*_+1_ + *E_k_*_,_*_t_*_+1_, *S_k_*_0_ = *n_k_, E_k_*_0_ = 1   - - Number of patients with infectivity at time *t*: *I_kt_*      - - Daily secondary infection rate of patient *i* (*i* = 1, …, *I_kt_*) with infectivity at time *t*    where **x***_it_* is defined as a covariate vector, including a time-dependent covariate of treatment, of patient *i* with infectivity at time *t*  Daily secondary infection rate in household *k* at time *t*:    Likelihood:   |
| --- |

**Table S1.** Performance of modified Reed-Frost model and Poisson regression model.

| **Influenza Type A** |  |  |  |
| --- | --- | --- | --- |
|  |  |  |  |
| Modified Reed-Frost model |  |  |  |
|  |  | Odds ratio (95% CI) | *P* value |
| Treatment | Peramivir | 0.32 (0.21, 0.47) | <0.0001 |
|  | Oseltamivir | 0.51 (0.37, 0.72) | <0.0001 |
|  | Zanamivir | 0.32 (0.21, 0.48) | <0.0001 |
|  | Laninamivir | 0.43 (0.28, 0.66) | <0.0001 |
|  | Untreated | --- | --- |
| Age | | 0.98 (0.97, 0.99) | 0.0002 |
| Time from onset to start of treatment | | 1.01 (1.00, 1.02) | 0.0205 |
| Influenza vaccination in the same season | | 1.14 (0.90, 1.45) | 0.2730 |
| Likelihood ratio test: *P* < 0.0001 (chi-squared value of 100.60 with 7 degrees of freedom) | | | |
|  |  |  |  |
| Poisson Regression Model |  |  |  |
|  |  | Risk ratio (95% CI) | *P* value |
| Treatment | Peramivir | 0.83 (0.53, 1.31) | 0.4263 |
|  | Oseltamivir | 1.23 (0.82, 1.85) | 0.3172 |
|  | Zanamivir | 0.80 (0.51, 1.27) | 0.3417 |
|  | Laninamivir | --- | --- |
| Age | | 0.99 (0.97, 1.00) | 0.0090 |
| Time from onset to start of treatment | | 1.01 (1.00, 1.02) | 0.0642 |
| Influenza vaccination in the same season | | 1.10 (0.83, 1.46) | 0.5139 |
| Likelihood ratio test: *P* < 0.0001 (chi-squared value of 29.66 with 6 degrees of freedom) | | | |
|  |  |  |  |
| **Influenza Type B** |  |  |  |
|  |  |  |  |
| Modified Reed-Frost model |  |  |  |
|  |  | Odds ratio (95% CI) | *P* value |
| Treatment | Peramivir | 1.70 (0.78, 3.71) | 0.1847 |
|  | Oseltamivir | 2.46 (1.21, 4.99) | 0.0130 |
|  | Zanamivir | 1.12 (0.59, 2.15) | 0.7280 |
|  | Laninamivir | 0.95 (0.38, 2.35) | 0.9107 |
|  | Untreated | --- | --- |
| Age | | 0.99 (0.97, 1.01) | 0.4684 |
| Time from onset to start of treatment | | 1.02 (1.01, 1.03) | 0.0027 |
| Influenza vaccination in the same season | | 0.92 (0.61, 1.38) | 0.6800 |
| Likelihood ratio test: *P* = 0.0086 (chi-squared value of 18.87 with 7 degrees of freedom) | | | |
|  |  |  |  |
| Poisson Regression Model |  |  |  |
|  |  | Risk ratio (95% CI) | *P* value |
| Treatment | Peramivir | 1.32 (0.58, 3.01) | 0.5151 |
|  | Oseltamivir | 2.08 (0.95, 4.56) | 0.0678 |
|  | Zanamivir | 1.00 (0.49, 2.02) | 0.9979 |
|  | Laninamivir | --- | --- |
| Age | | 1.00 (0.97, 1.02) | 0.7375 |
| Time from onset to start of treatment | | 1.01 (1.00, 1.02) | 0.0184 |
| Influenza vaccination in the same season | | 1.04 (0.67, 1.61) | 0.8642 |
| Likelihood ratio test: *P* = 0.0498 (chi-squared value of 12.60 with 6 degrees of freedom) | | | |

**Table S2.** Demographic characteristics of index patients infected with influenza A/H1pdm and A/H3.

|  |  | **Neuraminidase treatment** | | | | |
| --- | --- | --- | --- | --- | --- | --- |
| **Influenza Subtype** | **Variable** | **Peramivir** | **Oseltamivir** | **Zanamivir** | **Laninamivir** | **Untreated** |
| **A/H1pdm** | Number of index patients | 120 | 94 | 56 | 43 | 8 |
|  | Season, n (%) |  |  |  |  |  |
|  | 2010-2011 | 24 (20.0) | 23 (24.5) | 14 (25.0) | 17 (39.5) | 2 (25.0) |
|  | 2011-2012 | 0 | 0 | 0 | 0 | 0 |
|  | 2012-2013 | 0 | 0 | 0 | 0 | 0 |
|  | 2013-2014 | 37 (30.8) | 18 (19.1) | 13 (23.2) | 13 (30.2) | 3 (37.5) |
|  | 2014-2015 | 0 | 1 (1.1) | 0 | 0 | 0 |
|  | 2015-2016 | 59 (49.2) | 52 (55.3) | 29 (51.8) | 13 (30.2) | 3 (37.5) |
|  | Male, n (%) | 53 (44.2) | 57 (60.6) | 29 (51.8) | 20 (46.5) | 7 (87.5) |
|  | Age, years |  |  |  |  |  |
|  | n | 120 | 94 | 56 | 43 | 8 |
|  | Mean (SD) | 28.5 (19.2) | 13.1 (17.0) | 14.1 (11.5) | 19.5 (12.7) | 20.3 (18.6) |
|  | Median (min., max.) | 28.5 (2, 75) | 5.0 (0, 59) | 10.0 (4, 55) | 15.0 (5, 47) | 20.0 (0, 41) |
|  | Number of uninfected people per family at onset^a^ |  |  |  |  |  |
|  | n | 120 | 94 | 56 | 43 | 8 |
|  | Mean (SD) | 2.6 (1.1) | 2.9 (0.8) | 2.9 (1.1) | 3.0 (0.9) | 2.8 (1.4) |
|  | Median (min., max.) | 3.0 (0, 6) | 3.0 (1, 6) | 3.0 (0, 6) | 3.0 (1, 5) | 2.0 (1, 5) |
|  | Presence of influenza during previous season, n (%) | 11 (9.2) | 11 (11.7) | 5 (8.9) | 3 (7.0) | 0 |
|  | Influenza vaccination in the previous season, n (%) | 62 (51.7) | 42 (44.7) | 33 (58.9) | 21 (48.8) | 3 (37.5) |
|  | Influenza vaccination in the same season, n (%) | 54 (45.0) | 34 (36.2) | 27 (48.2) | 20 (46.5) | 2 (25.0) |
|  | Body temperature at first visit, °C |  |  |  |  |  |
|  | n | 120 | 89 | 56 | 41 | 8 |
|  | Mean (SD) | 38.24 (0.83) | 38.22 (1.01) | 38.20 (0.85) | 38.16 (0.82) | 37.63 (0.72) |
|  | Time from onset to start of treatment, days |  |  |  |  |  |
|  | n | 120 | 93 | 56 | 43 | NA |
|  | Mean (SD) | 0.8 (0.7) | 0.8 (0.7) | 0.8 (0.6) | 0.7 (0.6) | NA |
|  | Median (min., max.) | 0.7 (0, 3) | 0.7 (0, 3) | 0.7 (0, 3) | 0.7 (0, 3) | NA |
|  | Time from start of treatment to disappearance of fever, days |  |  |  |  |  |
|  | n | 108 | 84 | 54 | 39 | NA |
|  | Mean (SD) | 0.9 (0.7) | 1.2 (1.2) | 1.4 (1.4) | 1.0 (0.8) | NA |
|  | Median (min., max.) | 0.8 (0, 4) | 0.9 (−2, 6) | 0.9 (−1, 6) | 0.9 (0, 4) | NA |
|  |  |  |  |  |  |  |
| **A/H3** | Number of index patients | 258 | 229 | 192 | 127 | 19 |
|  | Season, n (%) |  |  |  |  |  |
|  | 2010-2011 | 13 (5.0) | 39 (17.0) | 24 (12.5) | 29 (22.8) | 4 (21.1) |
|  | 2011-2012 | 55 (21.3) | 52 (22.7) | 73 (38.0) | 20 (15.7) | 4 (21.1) |
|  | 2012-2013 | 77 (29.8) | 64 (27.9) | 28 (14.6) | 17 (13.4) | 3 (15.8) |
|  | 2013-2014 | 22 (8.5) | 21 (9.2) | 9 (4.7) | 10 (7.9) | 1 (5.3) |
|  | 2014-2015 | 86 (33.3) | 50 (21.8) | 55 (28.6) | 50 (39.4) | 7 (36.8) |
|  | 2015-2016 | 5 (1.9) | 3 (1.3) | 3 (1.6) | 1 (0.8) | 0 |
|  | Male, n (%) | 127 (49.2) | 122 (53.3) | 81 (42.2) | 66 (52.0) | 11 (57.9) |
|  | Age, years |  |  |  |  |  |
|  | n | 258 | 229 | 192 | 126 | 18 |
|  | Mean (SD) | 30.4 (18.9) | 12.6 (16.6) | 14.0 (11.7) | 15.3 (12.7) | 19.6 (16.0) |
|  | Median (min., max.) | 31.0 (2, 88) | 5.0 (0, 69) | 10.0 (3, 65) | 10.0 (3, 57) | 12.0 (2, 47) |
|  | Number of uninfected people per family at onset^a^ |  |  |  |  |  |
|  | n | 258 | 229 | 192 | 127 | 19 |
|  | Mean (SD) | 2.5 (1.1) | 2.7 (0.9) | 2.8 (1.0) | 2.9 (0.9) | 3.1 (1.0) |
|  | Median (min., max.) | 3.0 (0, 5) | 3.0 (0, 6) | 3.0 (0, 6) | 3.0 (0, 6) | 3.0 (1, 5) |
|  | Presence of influenza during previous season, n (%) | 25 (9.7) | 35 (15.3) | 42 (21.9) | 39 (30.7) | 2 (10.5) |
|  | Influenza vaccination in the previous season, n (%) | 143 (55.4) | 127 (55.5) | 104 (54.2) | 73 (57.5) | 6 (31.6) |
|  | Influenza vaccination in the same season, n (%) | 131 (50.8) | 138 (60.3) | 88 (45.8) | 68 (53.5) | 5 (26.3) |
|  | Body temperature at first visit, °C |  |  |  |  |  |
|  | n | 258 | 225 | 191 | 126 | 18 |
|  | Mean (SD) | 38.05 (0.86) | 38.30 (0.82) | 38.12 (0.84) | 38.07 (0.86) | 37.31 (0.94) |
|  | Time from onset to start of treatment, days |  |  |  |  |  |
|  | n | 258 | 229 | 192 | 127 | NA |
|  | Mean (SD) | 0.7 (0.6) | 0.8 (0.7) | 0.8 (0.7) | 0.8 (0.6) | NA |
|  | Median (min., max.) | 0.6 (0, 4) | 0.7 (0, 4) | 0.7 (0, 3) | 0.7 (0, 2) | NA |
|  | Time from start of treatment to disappearance of fever, days |  |  |  |  |  |
|  | n | 227 | 214 | 187 | 120 | NA |
|  | Mean (SD) | 0.8 (0.7) | 1.0 (0.9) | 1.1 (0.9) | 1.1 (1.1) | NA |
|  | Median (min., max.) | 0.8 (−2, 5) | 0.8 (−1, 5) | 0.8 (−1, 5) | 0.8 (−1, 6) | NA |

^a^Not including index patient.

Abbreviations: max., maximum; min., minimum; NA, not applicable; SD, standard deviation.

**Table S3**. Summary of index and secondary patients by age and role in family.

| **Type A** | **Index patient** | | | | | | |
| --- | --- | --- | --- | --- | --- | --- | --- |
| **Secondary patient** | Child ≤6 y | Child 7–12 y | Child 13–18 y |  | Father | Mother | Grandparent |
| Sibling ≤6 y | 20 (6.47%) | 12 (3.82%) | 0 (0.00%) |  | 9 (7.56%) | 10 (5.65%) | 0 (0.00%) |
| Sibling 7–12 y | 9 (2.91%) | 12 (3.82%) | 4 (2.52%) |  | 2 (1.68%) | 5 (2.82%) | 0 (0.00%) |
| Sibling 13–18 y | 0 (0.00%) | 2 (0.64%) | 0 (0.00%) |  | 1 (0.84%) | 0 (0.00%) | 0 (0.00%) |
| Father | 15 (4.85%) | 7 (2.23%) | 2 (1.26%) |  | --- | 4 (2.26%) | 0 (0.00%) |
| Mother | 46 (14.89%) | 24 (7.64%) | 9 (5.66%) |  | 8 (6.72%) | --- | 0 (0.00%) |
| Grandparent | 0 (0.00%) | 0 (0.00%) | 0 (0.00%) |  | 0 (0.00%) | 1 (0.56%) | 0 (0.00%) |
| None | 219 (70.87%) | 257 (81.85%) | 144 (90.57%) |  | 99 (83.19%) | 157 (88.70%) | 1 (100.00%) |
| Total | 309 | 314 | 159 |  | 119 | 177 | 1 |
| **Type B** | **Index patient** | | | | | | |
| **Secondary patient** | Child ≤6 y | Child 7–12 y | Child 13–18 y |  | Father | Mother | Grandparent |
| Sibling ≤6 y | 16 (8.25%) | 9 (3.10%) | 0 (0.00%) |  | 2 (8.00%) | 3 (7.14%) | 0 (0.00%) |
| Sibling 7–12 y | 9 (4.64%) | 8 (2.76%) | 1 (1.32%) |  | 0 (0.00%) | 1 (2.38%) | 0 (0.00%) |
| Sibling 13–18 y | 2 (1.03%) | 0 (0.00%) | 1 (1.32%) |  | 1 (4.00%) | 0 (0.00%) | 0 (0.00%) |
| Father | 2 (1.03%) | 2 (0.69%) | 0 (0.00%) |  | --- | 3 (7.14%) | 0 (0.00%) |
| Mother | 10 (5.15%) | 11 (3.79%) | 1 (1.32%) |  | 0 (0.00%) | --- | 0 (0.00%) |
| Grandparent | 0 (0.00%) | 0 (0.00%) | 0 (0.00%) |  | 0 (0.00%) | 0 (0.00%) | 0 (0.00%) |
| None | 155 (79.90%) | 260 (89.66%) | 73 (96.05%) |  | 22 (88.00%) | 35 (83.33%) | 0 (0.00%) |
| Total | 194 | 290 | 76 |  | 25 | 42 | 0 |

Note: The average time interval between index and secondary infection cases depended on the influenza type (type A: 2.5–2.7 days, type B: 3.3–3.9 days), but not on the neuraminidase inhibitor.
